# Supplementary material for: Neutrophils Promote Glioblastoma Tumor Cell Migration after Biopsy
Source: Cells. 2022 Jul 14;11(14):2196. doi: 10.3390/cells11142196 (PMC9324761; doi:10.3390/cells11142196)
Supplement: Supplementary file 1 [file cells-11-02196-s001.zip › Figure S1.pdf]

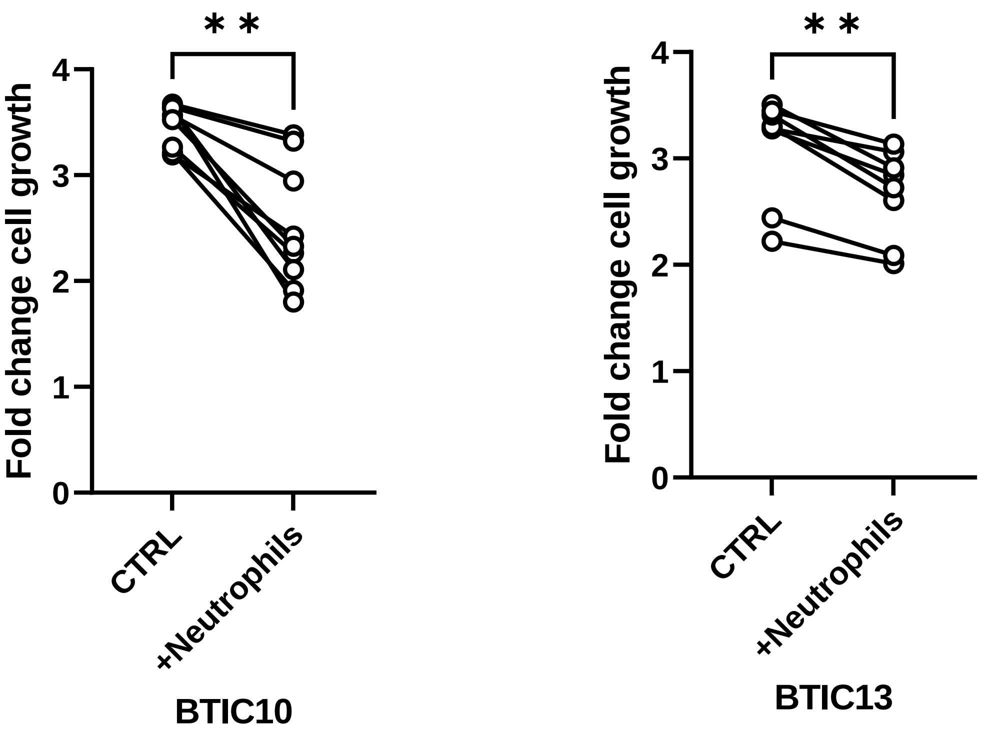

Supplementary Figure 1. Neutrophils do not promote cell growth of human glioblastoma tumor cells. BTIC10 and BTIC13 were seeded at a low confluency with or without neutrophils. The fold change of green intensity (tumor cells) at 22 hours compared to the first timepoint is depicted. Dots represent different neutrophil donors from two separate experiments. ** $p < 0.01$ in two-tailed Wilcoxon matched-pairs signed rank test.
